# Social conditions facilitate water conservation in a solitary bee

**DOI:** 10.1093/jisesa/ieae001

**Published:** 2024-02-03

**Authors:** Madeleine M Ostwald, Valentina A Venegas, Katja C Seltmann

**Affiliations:** Cheadle Center for Biodiversity & Ecological Restoration, University of California, Santa Barbara, CA, USA; Cheadle Center for Biodiversity & Ecological Restoration, University of California, Santa Barbara, CA, USA; Cheadle Center for Biodiversity & Ecological Restoration, University of California, Santa Barbara, CA, USA

**Keywords:** water balance, communal, bee, Melissodes tepidus timberlakei

## Abstract

Climatic stressors are important drivers in the evolution of social behavior. Social animals tend to thrive in harsh and unpredictable environments, yet the precise benefits driving these patterns are often unclear. Here, we explore water conservation in forced associations of a solitary bee (*Melissodes tepidus timberlakei* Cockerell, 1926) to test the hypothesis that grouping can generate synergistic physiological benefits in an incipient social context. Paired bees displayed mutual tolerance and experienced reduced water loss relative to singleton bees when exposed to acute low-humidity stress, with no change in activity levels. While the mechanism underlying these benefits remains unknown, social advantages like these can facilitate the evolution of cooperation among nonrelatives and offer important insights into the social consequences of climate change.

## Introduction

Group living can arise as an adaptive strategy for coping with environmental challenges. For insects, as small-bodied terrestrial organisms, conservation of body water is principal among these challenges (Hadley 1994). Temporary grouping is known to facilitate water conservation in insects, particularly during seasonal dry periods or vulnerable life history stages (Yoder and Grojean 1997, Klok and Chown 1999, Benoit et al. 2007). These same physiological benefits of grouping may also play a role in the evolution of more stable societies (Johnson 2021, Ostwald et al. 2022), although empirical evidence for these effects is scarce. Here, we artificially induce social conditions in a typically solitary bee (*Melissodes tepidus timberlakei* Cockerell, 1926) to test the hypothesis that grouping can generate water conservation benefits even in the absence of a phylogenetic history of social behavior.

Water availability shapes the distributions of social strategies by shaping the costs and benefits of grouping in a given environmental context (Purcell and Avilés 2008, Jetz and Rubenstein 2011, Lukas and Clutton-Brock 2017, La Richelière et al. 2022). Grouping can promote individual water conservation by generating a more favorable microclimate (Klok and Chown 1999, Danks 2002, Derhé et al. 2010), perhaps in part by increasing local humidity. For soil-nesting species, dehydration risks may be exacerbated by increased drought under climate change, which is causing rapid drying of soils in many regions (Berg and Sheffield 2018). These shifts in water availability and predictability will likely have profound but poorly understood consequences for animal social organization and social evolution (Blumstein et al. 2022, Ostwald et al. 2023).

Communal nesting strategies are often present at low levels in otherwise solitary populations (Ross and Matthews 1991, Michener 2007), providing a useful empirical context for examining the selective factors underlying transitions from solitary to group living. These societies often consist of unrelated individuals, wherein the mutualistic benefits of grouping compensate for the intrinsic costs of cooperating with nonkin (Clutton-Brock 2009, Ostwald et al. 2022). Artificial associations between normally solitary insects have provided powerful evidence for the emergent benefits of group living independent of an evolutionary history of sociality (Fewell and Page 1999, Holbrook et al. 2009). We investigated water conservation in a solitary bee (*M. tepidus timberlakei*) nesting in water-saturated soil. Communal nesting has not been described for *M. tepidus timberlakei*, but is known in the genus (Hurd and Linsley 1959) and its prevalence is likely underestimated (Ostwald et al. 2022). We exposed single and artificially paired bees to a low-humidity stress assay to explore social impacts on water balance.

## Materials and Methods

### Study Population, Nesting Biology, and Field Collections

We sampled bees from an aggregation of *M. tepidus timberlakei* nesting along the shoreline of a coastal lagoon on the University of California, Santa Barbara campus (34.410358 N, −119.850442 W). This nesting aggregation hosts several hundred nests annually and has been observed at this location since at least 2019 (Seltmann pers. obs). Nests are predominantly concentrated along a 40-m stretch of the lagoon shoreline, within 2 m of the water, and are found both in bare soil and beneath patches of Alkali Heath (*Frankenia salina* (Molina) I.M. Johnst). Nest initiation began in mid-May 2023, and flight activity continued through July 2023. We measured soil moisture approximately monthly throughout the nesting site using a TDR (time-domain reflectometry) soil moisture meter (FieldScout TDR 300, Spectrum Technologies, Inc., Aurora, IL) between June 2022 and May 2023 and found that the soil was consistently at its saturation point (52% volumetric water content).

We collected adult female *M. tepidus timberlakei* exiting their nests between late June and early July 2023. We transported bees to the lab within 1 h of collection, where they were weighed using a microbalance (0.001 g precision). All bees were paint-marked on the thorax to facilitate identification during data collection and to prevent resampling on subsequent days.

### Water Loss Assays

We used a gravimetric water loss assay to compare water loss in single and paired bees at roughly 0% humidity, at 24 ± 1 °C over 3 h. Bees were placed in 15 mL falcon tubes containing 5 mL of desiccant (indicator silica gel), from which they were separated by 2 mL of cotton. Bees were randomly assigned to a social condition treatment as either singletons (1 bee per tube) or pairs (2 bees per tube). We kept the bees in the 0% humidity tubes for 3 h before reweighing. We chose the 3-h experimental period following pilot assays that indicated this exposure time resulted in minimal mortality. We assumed that mass loss equaled water loss over this time interval (Hadley 1994) and estimated total water loss as the difference in mass before and after the assay. We estimated water loss as a percentage of total body mass as follows:


% body mass lost=[(initial mass−f inal mass) / (initial mass)]×100

Initial mass did not differ significantly by treatment (singleton mean = 0.045 g; paired mean = 0.045 g; *t*-test: *P *= 0.224). Additionally, to estimate the total body water content of *M. tepidus timberlakei* females, we dried a subset of 10 females in a drying oven for 3 days at 60 °C, and then subtracted dry mass from initial live mass. This metric was used only to contextualize our water loss estimates and was not included in our statistical analysis.

### Behavioral Observations

To estimate the activity level of bees as a possible predictor of water loss, we performed behavioral scan samples of each individual every 20 min throughout the 3-h assay. We recorded behaviors according to a standard ethogram and observed three unique behaviors during the assays: (i) idle, (ii) walking/climbing, and (iii) self-grooming. No social behaviors, including agonistic interactions, were observed. We estimated the proportion of active time as the number of scan samples in which an active behavior (walking/climbing) was recorded divided by the total number of scan samples. Following the assays, bees were rehydrated and returned to their nest site.

### Statistical Analysis

To understand how social condition influences water conservation, we fitted a linear regression model with absolute mass loss as the response variable. As predictor variables, we included factors we expected to influence water conservation: social condition (singleton vs. paired), initial body mass, the proportion of active time, and the interaction between the social condition and the proportion of active time. We confirmed that our model met assumptions of normality and homoscedasticity by evaluating Q-Q plots and plots of fitted values vs. residuals. We then performed a Type II ANOVA on the fitted model to evaluate the significance of our predictor values. We additionally performed a Wilcoxon test to test the hypothesis that social condition influences active time, i.e., if proximity to another bee stimulates activity. We excluded from our analysis any bees that died over the course of the assay (*N* = 7 of 67; 10.4%), as well as any bees that were paired with bees that died, resulting in a final sample size of 31 singleton bees and 28 paired bees (e.g., 14 pairs). Results are presented as mean ± standard error. All analyses were performed in R version 4.2.1 (R Core Team 2022).

## Results

**Fig. 1.**
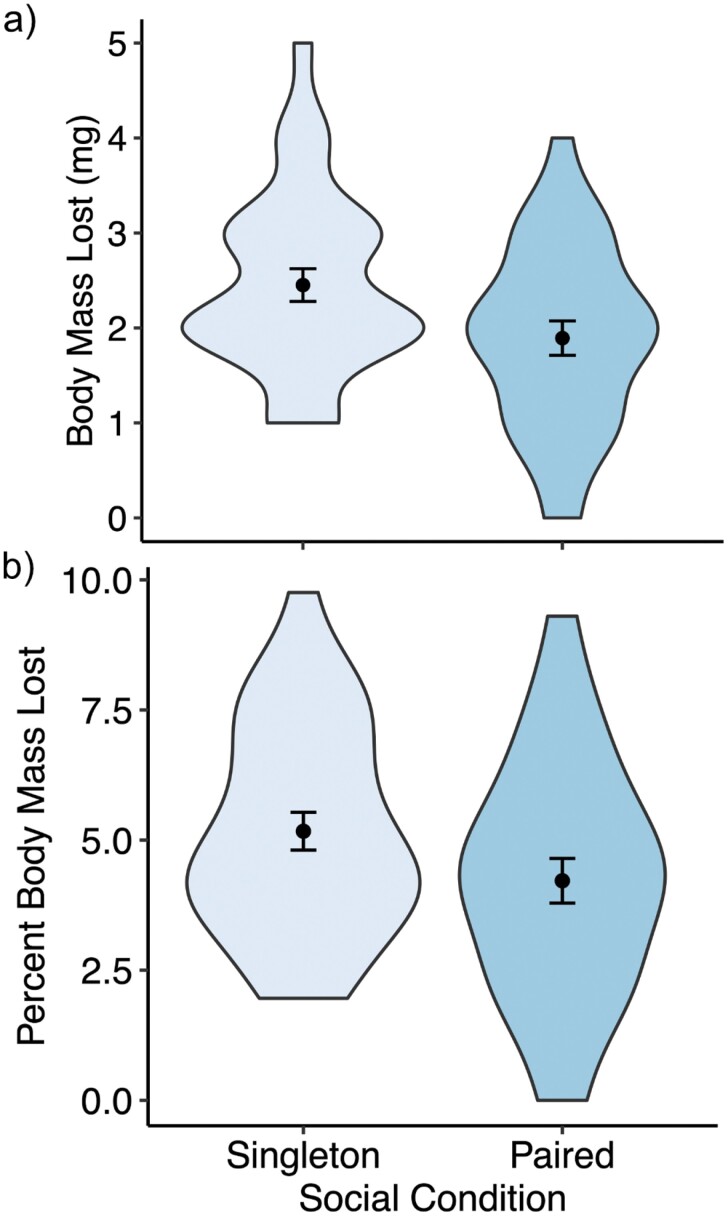
Water loss in singleton (*n* = 31) vs. paired (*n* = 28) bees estimated as the (a) absolute and (b) percent mass lost over 3 hours at 0% humidity. Singleton bees lost significantly more body mass than paired bees (ANOVA: *P* = 0.020). The width of the violin indicates the density of the data at a given value. The black dots and error bars show mean and standard error, respectively.

Social condition significantly predicted mass loss (*P *= 0.036, *R*^2^ = 0.111) at 0% humidity (Table 1; Fig. 1). Paired bees lost 1.892 ± 0.181 mg (4.22 ± 1.81%) of body mass, while singleton bees lost 2.452 ± 0.173 mg (5.17 ± 0.04%) of body mass. The proportion of active time also significantly predicted mass loss, but initial body mass did not (Table 1). Likewise, the social condition did not significantly influence the proportion of active time (Wilcoxon test: *P *= 0.100). Females sampled in our study contained 32.9 ± 2.37 mg (67.6 ± 4.89%) body water.

**Table 1.** Summary of ANOVA comparisons evaluating the effects of evaluated predictor variables (social condition, activity time, initial mass, and the interaction between social condition and activity time) on mass loss

	Sum Sq.	Df	*F*-value	*P*-value
Predictor				
Social condition (singleton vs. paired)	5.076 × 10^−6^	1	5.785	**0.020**
Proportion of time spent active	4.143 × 10^−6^	1	4.722	**0.034**
Initial mass	1.253 × 10^−6^	1	1.428	0.237
Social condition:Prop. active time	4.190 × 10^−6^	1	0.560	0.458
Residuals	4.651 × 10^−5^	53		

Bolded values indicate statistical significance (*P* < 0.05).

## Discussion

Group living fundamentally alters an organism’s experienced microclimate, potentially offering refuge from stressful environmental conditions (Yahav and Buffenstein 1991, Krause and Ruxton 2002, Gilbert et al. 2006, Jones and Oldroyd 2006, Boratynski et al. 2015). Here, we found support for our hypothesis that social conditions can facilitate water conservation in typically solitary bees. When exposed to low humidity, paired bees retained more water than bees kept alone, with no differences in activity level. These advantages of social living offer insights into the ecological conditions that can give rise to incipient communal societies.

Several mechanisms have been proposed to account for social water conservation advantages. These benefits have been described across arthropod taxa, including beetles (Yoder et al. 1992, Rasa 1997, Bong et al. 2018), cockroaches (Yoder and Grojean 1997), bed bugs (Benoit et al. 2007), woodlice (Derhé et al. 2010, Broly et al. 2014), and larval Lepidoptera (Willmer 1980, Klok and Chown 1999). These animals aggregate in groups of tens to thousands of individuals, greatly reducing their collective surface-area-to-volume ratio and thereby reducing evaporative water loss. Interestingly, we found similar benefits for groups of just 2 individuals. Paired bees in our study generally stood adjacent to one another rather than forming a tight huddle, indicating that their effective surface-area-to-volume ratios were not substantially changed in the social treatment. Instead, it may be that groups benefited from altered microclimates, perhaps through the creation of a humidified boundary layer via mutual transpiration (Benoit et al. 2007).

Bees in the social treatment experienced a modest but significant reduction in water loss relative to singletons in just 3 h at 0% humidity (paired bees: 5.75% of body water lost; singleton bees: 7.45% of body water lost). Over longer time periods in natural contexts, accumulated differences in water loss could feasibly lead to differential mortality and/or fitness outcomes for solitary vs. social individuals. Similarly, we found no effect of body size on water loss in our study, though longer exposures to low-humidity stress could reveal size effects on water balance, as in other systems (Hadley 1994, Addo-bediako et al. 2001). Importantly, our study design allowed for nondestructive sampling with minimal observed harm to study subjects. Bees were rehydrated and returned to their nest sites within 5 h of capture and observed foraging on subsequent days. Many common insect physiological stress assays are lethal or inflict severe sublethal injuries, limiting their usefulness for large-scale studies of nonmodel insect systems or for repeated sampling of individuals over time. Our study provides a template for future studies aimed at expanding our understanding of physiological stress responses in rare and declining bee species while minimizing impacts on source populations.

Under climate change, increasing drought will restrict soil moisture in many regions, with unknown consequences for the behavior and distributions of ground-nesting bees, many of which exhibit preferences for particular soil abiotic conditions (Cane 1991, Antoine and Forrest 2021). The low-humidity conditions in our study represent acute hygric stress and allow us to explore differential water loss attributable to social condition. Drought conditions, by contrast, would entail less extreme humidity stress but over longer time periods. How bees fare under humidity stress in natural conditions remains to be explored. Our study population of *M. tepidus timberlakei* nests in water-saturated soil, but ground-nesting bees vary enormously in their soil water content preferences (Cane 1991). In dry soils, cohabitating with other females could increase humidity in nesting tunnels and help mitigate the challenges of maintaining water homeostasis in dry soils.

Combined, our behavioral and physiological data suggest both tolerance for conspecifics as well as a physiological advantage of grouping in dry conditions. Future studies should clarify the mechanisms underlying this pattern and determine whether these benefits exist in natural contexts. Water loss increased significantly with active time, in line with previous studies (Lighton and Bartholomew 1988, Hadley 1994), yet paired bees were no more active than singleton bees, suggesting that proximity to another bee did not stimulate activity (e.g., avoidance). Furthermore, we did not observe any instances of aggression in pairs. The lack of aggressive or avoidant behaviors observed in our study mirrors observations of other communal and solitary bees (McConnell-Garner and Kukuk 1997, Packer 2006) and indicates a capacity for mutual tolerance of unrelated conspecifics. Contexts like these that combine behavioral plasticity with selective advantages for social individuals may broadly resemble conditions at the evolutionary origins of group living.

## Data Availability

All data and associated code are available at https://doi.org/10.5281/zenodo.8237366.
